# Complete and durable remission in a patient with brain metastasis from gastric cancer treated with SRS followed by RC48 plus camrelizumab after progression on chemo-immunotherapy: a case report and literature review

**DOI:** 10.3389/fimmu.2026.1883629

**Published:** 2026-09-03

**Authors:** Xiao Liu, Baozhen Ma, Mengjiao Yang, Lingdi Zhao

**Affiliations:** 1 Radiotherapy department, the Affiliated Cancer Hospital of Zhengzhou University & Henan Cancer Hospital, Zhengzhou, China; 2 Immunotherapy department, the Affiliated Cancer Hospital of Zhengzhou University & Henan Cancer Hospital, Zhengzhou, China

**Keywords:** brain metastasis, gastric cancer, HER2-low, PD-1 inhibitor, RC48, stereotactic radiosurgery

## Abstract

**Background:**

Brain metastasis from gastric cancer carries a dismal prognosis, with median overall survival of only 3–6 months. Patients who progress on immune checkpoint inhibitor (ICI)-containing regimens have limited salvage options. HER2-low expression (IHC 1+ or IHC 2+/FISH-negative), historically excluded from anti-HER2 therapy, has now emerged as a novel therapeutic target for antibody-drug conjugates (ADCs) such as RC48 (disitamab vedotin).

**Case summary:**

A 52-year-old male with HER2-low (IHC 2+/FISH-negative), PD-L1 CPS 5, microsatellite-stable (MSS) advanced gastric cancer received first-line XELOX plus camrelizumab, achieving a partial response (PR) followed by progression with new brain metastases. After stereotactic radiosurgery (SRS) for a solitary brain lesion, he received RC48 plus camrelizumab for eight months, achieving PR. Treatment was discontinued due to grade 2 pneumonitis (managed without corticosteroids). Complete response (CR) was achieved during the follow-up. The patient has been off therapy over three years and remains disease-free.

**Literature review:**

We review the current evidence for HER2-low gastric cancer treatment, ADC plus ICI combinations, the potential immunomodulatory role of radiotherapy (including the abscopal effect) as it may contribute to systemic antitumor responses when combined with ICIs, and treatment-free remission following ICI discontinuation.

**Conclusion:**

This is the first report of durable, treatment-free survival over 36 months in an ICI-refractory, HER2-low gastric cancer patient with brain metastasis suggests that RC48 can reverse PD-1 resistance, SRS may prime systemic antitumor immunity, and finite-duration ADC plus ICI therapy can establish long-lasting immunological memory.

## Introduction

Globally, gastric cancer remains the fifth most common malignancy and the fourth leading cause of cancer-related mortality, with over one million new cases annually (1). Despite advances in perioperative chemotherapy and targeted therapies, the prognosis for patients with advanced or metastatic gastric cancer remains poor, with median overall survival (OS) of less than 12 months following first-line treatment failure (2).

Human epidermal growth factor receptor 2 (HER2) positivity, defined as immunohistochemistry (IHC) 3+ or IHC 2+ with fluorescence *in situ* hybridization (FISH) amplification, occurs in approximately 15-20% of gastric cancer patients and predicts response to trastuzumab (3). However, 40-60% of gastric cancer patients exhibit HER2-low expression (IHC 1+ or IHC 2+/FISH-negative), a subgroup historically excluded from anti-HER2 therapy (4). The advent of antibody-drug conjugates (ADCs) has revolutionized this paradigm. Unlike traditional monoclonal antibodies, ADCs possess a potent cytotoxic payload that can be internalized into HER2-expressing tumor cells and exert a “bystander killing effect” on adjacent HER2-negative cells, enabling efficacy even in low-expression settings (5). RC48 (disitamab vedotin) is a novel ADC composed of an anti-HER2 antibody conjugated to monomethyl auristatin E (MMAE). The pivotal phase II RC48-C008 study demonstrated an objective response rate (ORR) of 24.8% and median OS of 7.9 months in HER2-expressing advanced gastric cancer patients who had failed at least two prior lines of therapy (6). Subsequently, RC48 received conditional approval in China for HER2-low advanced gastric cancer after at least two prior chemotherapy regimens.

Immune checkpoint inhibitors (ICIs) targeting the PD-1/PD-L1 axis have become first-line standard of care for advanced gastric cancer, particularly for patients with PD-L1 combined positive score (CPS) ≥ 5 (7, 8). However, primary or acquired resistance to ICIs remains a major clinical challenge, and effective salvage therapies after ICI progression are scarce (9). Preclinical evidence suggests that ADCs and ICIs may exert synergistic antitumor effects. ADC-induced immunogenic cell death (ICD) releases damage-associated molecular patterns (DAMPs) and tumor antigens, promoting dendritic cell maturation and CD8+ T-cell priming, thereby converting “cold” tumors into “hot” tumors susceptible to PD-1 blockade (10, 11).

Brain metastasis from gastric cancer is a relatively rare but devastating event, occurring in approximately 3-5% of patients, with median OS ranging from 3 to 6 months even after radiotherapy (12, 13). Patients with brain metastases are systematically excluded from most clinical trials of ADCs or ICIs, creating a critical knowledge gap. Herein, we report an unusual case of a patient with HER2-low, PD-L1 CPS 5, MSS advanced gastric cancer who developed brain metastasis after progression on first-line chemo-immunotherapy. Following SRS for the solitary brain lesion, the patient was treated with RC48 plus camrelizumab for eight months until January 2023, when treatment was discontinued due to grade 2 pneumonitis. A complete response (CR) was subsequently achieved in July 2024, and the patient has remained in CR for over 21 months. We also provide a literature review contextualizing this case within the current evidence base.

## Case presentation

### Patient information and initial diagnosis

**Figure 1:**
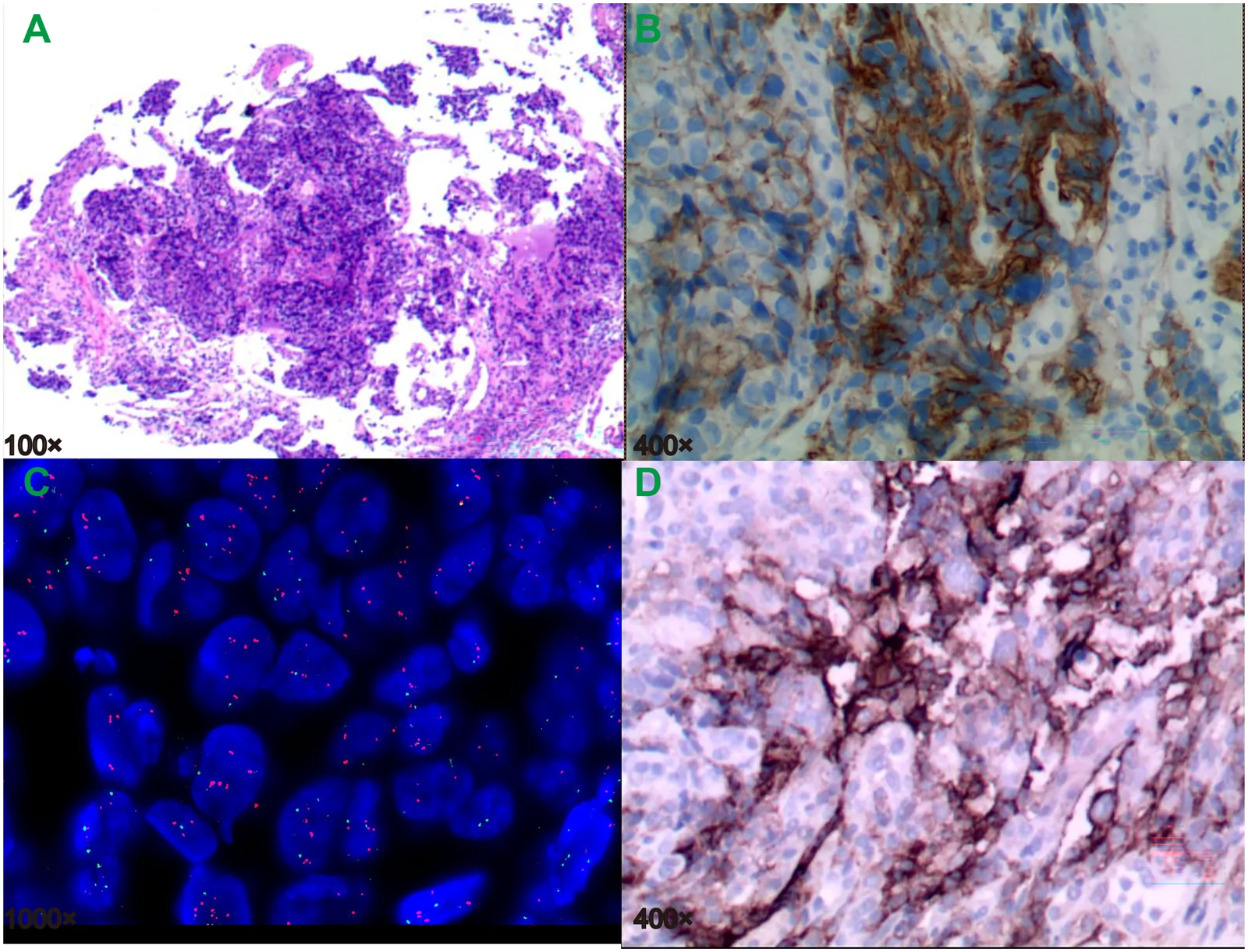
Pathological characterization of the primary tumor. **(A)** Hematoxylin and eosin (H&E) staining showing poorly differentiated adenocarcinoma. **(B)** HER2 immunohistochemistry (IHC) demonstrating 2+ membranous staining. **(C)** HER2 fluorescence *in situ* hybridization (FISH) showing no amplification (HER2/CEP17 ratio <2.0). **(D)** PD-L1 IHC with a combined positive score (CPS) of 5.

A 52-year-old male presented to our hospital in February 2021 with a two-month history of progressive epigastric fullness and dull pain, which had initially begun in October 2020. He had no significant past medical history other than an active hepatitis C virus (HCV) infection. Upper gastrointestinal endoscopy revealed an infiltrative, ulcerative lesion extending from the lower esophagus (35–44 cm from the incisors) to the gastric cardia and the lesser curvature/posterior wall of the upper gastric body. Biopsy confirmed poorly differentiated adenocarcinoma (**Figure 1A**). Immunohistochemistry (IHC) demonstrated HER2 expression of 2+, with fluorescence *in situ* hybridization (FISH) negative for amplification (**Figures 1B, C**). The tumor was microsatellite stable (MSS) with a programmed death-ligand 1 (PD-L1) combined positive score (CPS) of 5 (**Figure 1D**).

**Figure 2:**
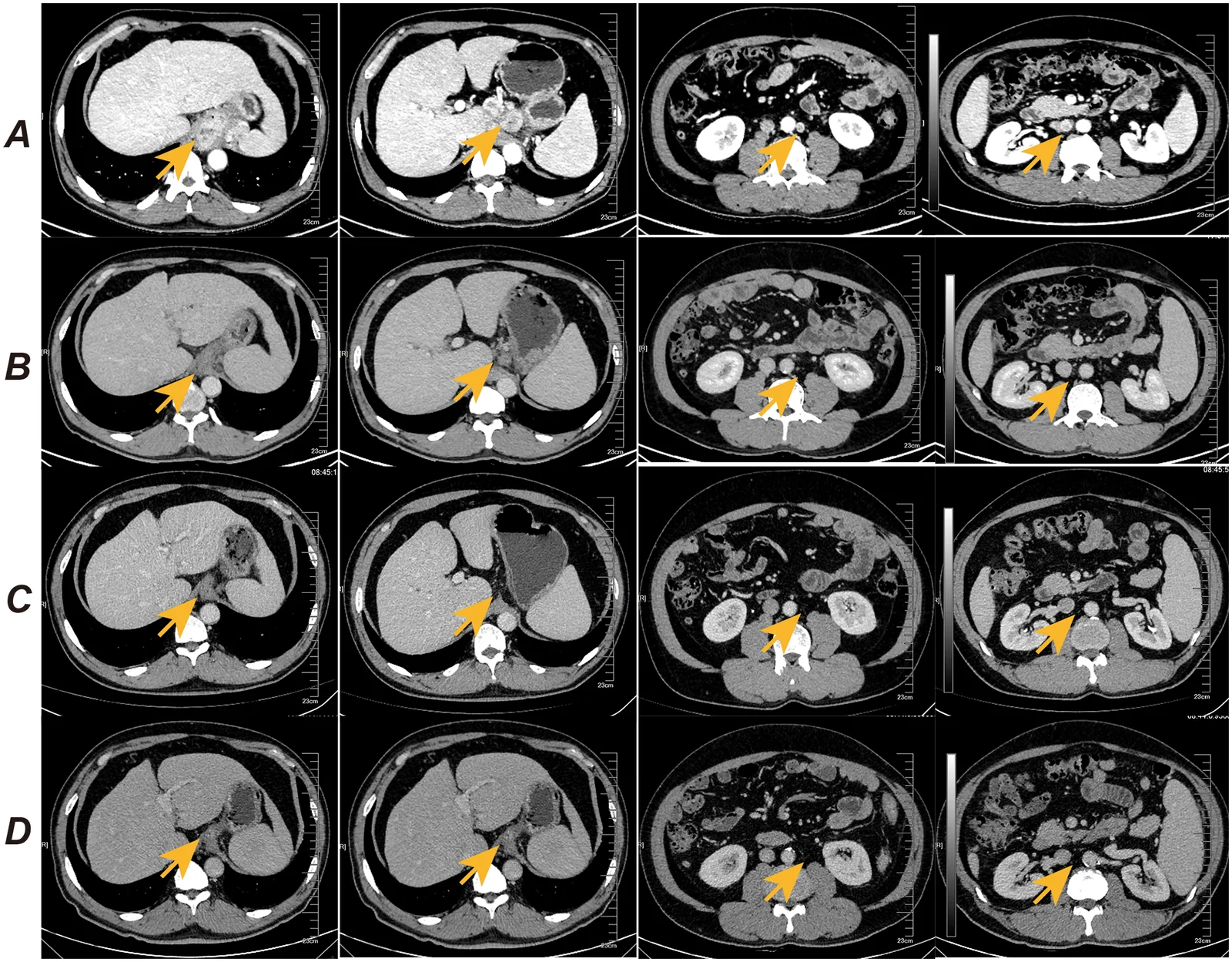
Radiographic evolution of tumor burden during the treatment course. **(A)** Baseline CT scans showing the primary esophagogastric junction tumor, perigastric and retroperitoneal lymph nodes. **(B)** After two cycles of XELOX chemotherapy, showing a partial response with significant tumor reduction. **(C)** At the time of brain metastasis diagnosis (May 2022), showing stable extracranial disease without systemic progression. **(D)** Most recent follow-up (October 2025), demonstrating a sustained complete response with complete resolution of all lesions.

Contrast-enhanced computed tomography (CT) of the abdomen showed thickening of the distal esophageal and gastric cardia wall, consistent with an esophagogastric junction carcinoma, along with multiple enlarged lymph nodes in the perigastric and retroperitoneal regions. The clinical stage was cT4N+M1 (**Figure 2A**). Following multidisciplinary team discussion, first-line chemo-immunotherapy was recommended.

### First-line therapy and progression

The patient had an active hepatitis C virus infection at diagnosis. Therefore, antiviral therapy for HCV was initiated concurrently with chemotherapy. From March to April 2021, the patient received two cycles of the XELOX regimen (capecitabine plus oxaliplatin) and achieved a partial response (PR) (**Figure 2B**). Subsequently, HCV RNA became undetectable. He then received four cycles of XELOX plus camrelizumab (200 mg intravenously every three weeks), followed by camrelizumab maintenance therapy.

**Figure 3:**
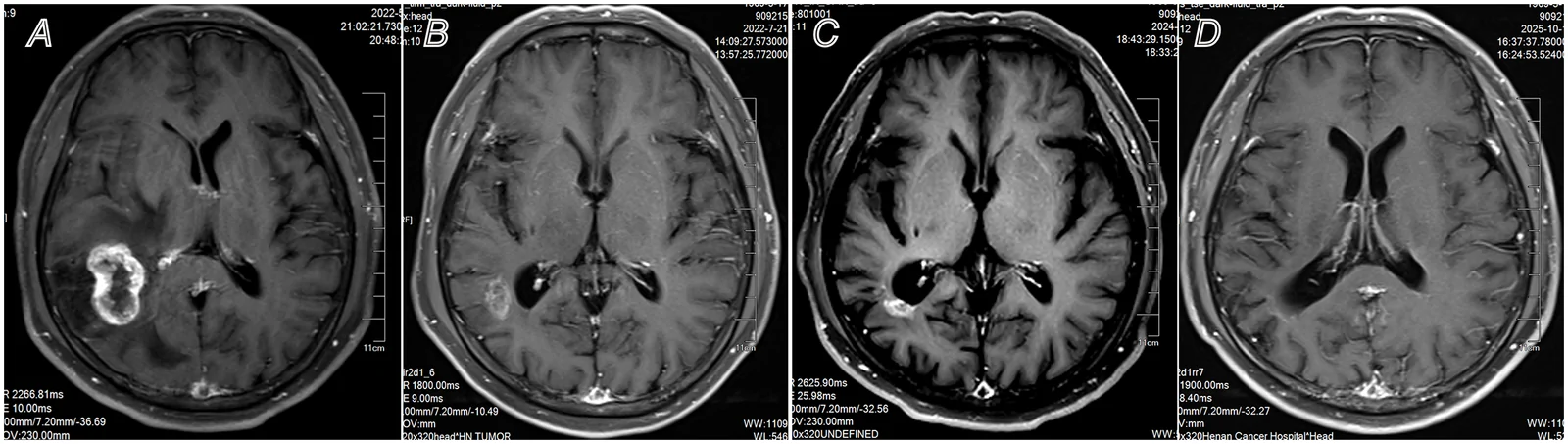
Intracranial response to stereotactic radiosurgery and subsequent RC48 plus camrelizumab therapy. **(A)** Baseline contrast-enhanced T1-weighted MR image reveals a nodular lesion exhibiting heterogeneous enhancement in the right temporo-occipital lobe, associated with surrounding peritumoral edema. The tumor measured 34 mm in maximum diameter (34 × 20 mm). **(B)** T1-weighted contrast-enhanced MRI obtained after SRS and two cycles of RC-48 plus camrelizumab, demonstrating a reduction in the enhancing nodule in the right temporo-occipital lobe, with decreased peritumoral edema. Lesion size: 13 × 20 mm. **(C)** Contrast-enhanced brain MRI at treatment cessation revealed a heterogeneously enhancing nodule in the right temporo-occipital lobe (longest diameter: ~20 mm) with peritumoral edema, showing no significant change from prior scans. **(D)** Contrast-enhanced T1-weighted MR image at the last follow-up (October 2025), the abnormal signal in the right temporo-occipital periventricular area was substantially decreased relative to prior scans, and PWI demonstrated no evidence of hyperperfusion.

In May 2022, approximately 14 months after starting first-line therapy, the patient experienced persistent headache. Brain magnetic resonance imaging (MRI) revealed metastatic lesions located in the right temporal lobe and right occipital lobe (**Figure 3A**). There was no evidence of systemic progression on concurrent CT imaging (**Figure 2C**).

### Stereotactic radiosurgery for brain metastases and salvage therapy

The patient received SRS for the brain metastases, with a total dose of 30 Gy delivered in 5 fractions. Following SRS, his headache resolved completely. Subsequent systemic treatment was switched to a combination of RC48 (disitamab vedotin) at a dose of 2.5 mg/kg intravenously every three weeks, plus camrelizumab 200 mg intravenously every three weeks. After two cycles of RC48 plus camrelizumab (August 2022), restaging imaging showed stable extracranial disease and marked shrinkage of intracranial lesions, consistent with a PR (**Figures 2D**, **3B**). The patient continued the same combination for a total treatment duration of eight months. In January 2023, the patient developed grade 2 pneumonitis according to the Common Terminology Criteria for Adverse Events (CTCAE), characterized by dry cough and patchy ground-glass opacities on chest CT. He did not receive corticosteroids; instead, symptomatic management with antitussive agents and close monitoring was provided. The pneumonitis gradually resolved over six weeks without sequelae.

### Follow-up and sustained remission

**Figure 4:**
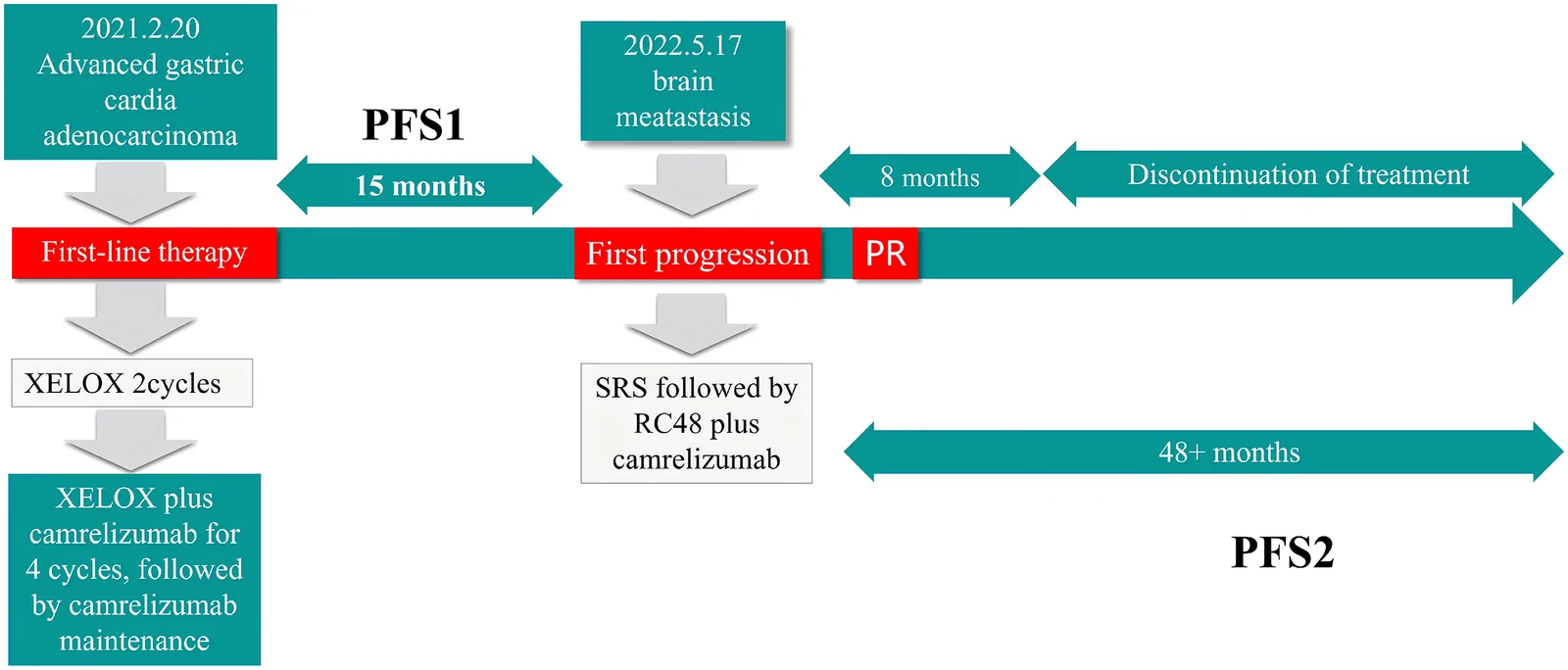
Schematic timeline.

Since stopping all anti-tumor therapy in January 2023, the patient has remained under regular surveillance with CT and MRI scans performed every four to six months. In January 2024—one year after treatment discontinuation—brain MRI demonstrated further reduction of the residual intracranial lesions, with no evidence of local tumor activity on functional imaging (**Figure 3C**). By July 2024, complete resolution of intracranial lesions was confirmed, which has been sustained through the last follow-up in October 2025 (**Figures 2D**, **3D**), with an progression-free survival exceeding 47 months from the start of RC48 plus camrelizumab and over 38 months of treatment-free remission until present. His Eastern Cooperative Oncology Group performance status is 0, and he reports a normal quality of life. **Figure 4** provides a schematic timeline of the overall clinical course.

## Literature review

### Search strategy

To contextualize this case, we performed a comprehensive literature search of PubMed and Web of Science databases from January 2018 to April 2026, using the following keywords: “gastric cancer,” “HER2-low,” “RC48,” “disitamab vedotin,” “antibody-drug conjugate,” “immune checkpoint inhibitor,” “PD-1,” “brain metastasis,” “stereotactic radiosurgery,” and “abscopal effect”. Relevant original articles, clinical trials, case series, and prior case reports were reviewed.

### ADC plus immune checkpoint inhibitor combinations

The combination of ADCs with ICIs represents one of the most promising therapeutic strategies in HER2-expressing gastric cancer, particularly in the post-immunotherapy refractory setting. The mechanistic rationale for this combination is compelling. ADC-induced ICD releases DAMPs and tumor antigens, promoting dendritic cell maturation and CD8+ T-cell priming (10, 11). ADCs can also deplete immunosuppressive cells (Tregs, MDSCs) within the tumor microenvironment and upregulate PD-L1 on surviving tumor cells, rendering them more susceptible to PD-1 blockade (14). This synergistic interplay between ADC-mediated cytotoxicity and ICI-mediated T-cell reactivation forms the biological foundation for combination strategies. The most definitive evidence for RC48 plus ICI combinations comes from the phase I study, which prospectively evaluated RC48 plus toripalimab in HER2-expressing advanced solid tumors, including a gastric/gastroesophageal junction cancer cohort of 56 patients. The confirmed ORR was 43%, the disease control rate (DCR) was 90.5%, and median progression-free survival (PFS) was 6.9 months (15).

Complementing these prospective data, a subsequent real-world multicenter retrospective study by Yan et al. directly compared RC48 plus a PD-1 inhibitor versus RC48 monotherapy in patients with HER2-expressing advanced gastric cancer who had failed at least two prior lines of therapy. The combination group (n=36) achieved significantly higher ORR (41.7% vs. 9.5%, p=0.011) and longer median OS (13.2 vs. 7.1 months, p=0.04) compared to RC48 monotherapy (n=21) (16). These real-world data strongly support the clinical utility of RC48 plus ICI combinations, particularly in the ICI-refractory setting.

Trastuzumab deruxtecan (T-DXd, DS-8201) has also been investigated in combination with ICIs. The DESTINY-Gastric03 study, a Phase Ib/II trial, demonstrated that in the HER2-overexpressing gastric cancer cohort, treatment with DS-8201 in combination with pembrolizumab yielded an ORR of 63% and a median PFS of 10 months (17). The RCTS study, a Phase II trial, evaluated the combination of RC48, S-1, and tislelizumab in HER2-overexpressing advanced gastric cancer. In the 57 patients enrolled, the ORR was 89.5%, and the median OS was 31.9 months (18). A head-to-head comparison between RC48 and T-DXd in the ICI combination setting is not yet available, and agent selection is currently guided by differences in payload (MMAE for RC48 vs. DXd for T-DXd), drug-to-antibody ratio, toxicity profiles, and regional availability.

To provide a concise overview of the available evidence, we have summarized the key studies evaluating ADC plus ICI combinations in HER2-expressing gastric cancer in **Table 1**. Collectively, these data highlight that the addition of an ICI to ADC therapy not only improves response rates and survival outcomes but also appears to re-sensitize tumors that have progressed on prior immunotherapy—a phenomenon that is central to the novelty of our case.

**Table 1:** Summary of key studies evaluating ADC plus immune checkpoint inhibitor in HER2-expressing advanced gastric cancer.

Study	Design	Regimen	Population	N	ORR	Median PFS (months)	Median OS (months)
Wang et al. (15)	Phase I	RC48+ toripalimab	HER2-expressing	30	43%	6.2	16.8
Yan et al. (16)	Retrospective	RC48 + PD-1 inhibitor	HER2 overexpressing	36	41.7%	5.8	13.2
DESTINY-Gastric03 (17)	Phase Ib/II	DS8201+K	HER2 overexpressing	41	63%	10	20
DESTINY-Gastric03 (19)	Phase Ib/II	DS8201 + 5-Fu+K	HER2 overexpressed	32	75%	9.8	15.7
RCTS (18)	Phase II	RC48 + tislelizumab + S-1	HER2-overexpressing	57	89.5%	13.8	31.9

### Brain metastasis in gastric cancer

Brain metastasis (BM) from gastric cancer occurs in approximately 3-5% of patients, and the prognosis following BM diagnosis is exceedingly poor, with median OS ranging from 3 to 6 months (12, 13). Local therapies remain the mainstay of treatment. Whole-brain radiotherapy provides symptomatic relief but offers limited survival benefit (median OS 3–4 months). SRS is preferred for patients with limited (≤3) and small (≤3 cm) brain metastases, achieving local control rates of 70–90% and median OS of 6–12 months in selected patients (20).

Data on systemic therapy for brain metastases from gastric cancer are extremely limited. Traditional chemotherapeutic agents have poor blood-brain barrier penetration. Emerging data from HER2-positive breast cancer suggest that ADCs such as DS-8201 have robust intracranial activity, with intracranial ORR of 73-79% in patients with active brain metastases (21). Extrapolating from these data, it is plausible that RC48 may also have intracranial activity in gastric cancer patients with brain metastases, although prospective data are lacking.

### Treatment-free remission and immunological memory

Treatment-free remission (TFR) – durable response off-therapy – has been increasingly recognized in patients treated with ICIs. In melanoma, approximately 20-30% of patients who achieve CR with nivolumab plus ipilimumab can discontinue treatment without relapse (22). In non-small cell lung cancer, 15-20% of patients who complete two years of pembrolizumab remain progression-free off-therapy (23). TFR is thought to be mediated by long-lived memory T cells, including central memory T cells (TCM), effector memory T cells (TEM), and tissue-resident memory T cells (TRM) (24). The establishment of immunological memory typically requires sustained antigen exposure during initial treatment, adequate CD4+ T-cell help, and an appropriate cytokine milieu supporting memory T-cell homeostasis. In gastric cancer, data on TFR are extremely limited. Most clinical trials have continued treatment until progression, precluding analysis of treatment discontinuation. To our knowledge, our case represents one of the longest reported TFR durations (over 36 months) in a patient with advanced gastric cancer treated with immunotherapy-containing regimen.

### Immune-related adverse events and treatment outcomes

The development of immune-related adverse events (irAEs) is associated with improved clinical outcomes in patients treated with ICIs. A meta-analysis of 45 studies involving over 10,000 patients found that irAE occurrence was associated with a 40% reduction in the risk of death (HR 0.60, 95% CI 0.54-0.66) (25). Specifically, ICI-related pneumonitis has been linked to superior survival outcomes in non-small cell lung cancer (26). Importantly, withholding corticosteroids for grade 2 pneumonitis in the absence of significant hypoxemia may help preserve ongoing antitumor immunity, as systemic corticosteroids can suppress T-cell function and may abrogate the clinical benefit of immunotherapy (27). While guidelines generally recommend corticosteroids for grade ≥2 pneumonitis, our case suggests that close observation without steroids is a viable strategy for select patients with mild, self-limited symptoms.

## Discussion

We report a case of a patient with HER2-low, PD-L1 CPS 5, MSS advanced gastric cancer who progressed on first-line chemo-immunotherapy and developed brain metastasis. Following SRS, the patient received RC48 plus camrelizumab for eight months. Treatment was then discontinued due to grade 2 pneumonitis, which was managed without corticosteroids. CR was achieved and has been sustained for over 36 months off all anti-tumor therapy. To our knowledge, this is the first report of durable, treatment-free CR exceeding three years in this clinical scenario.

A striking feature of this case is that the patient clearly progressed on camrelizumab-containing therapy yet responded when camrelizumab was combined with RC48. This observation aligns with the real-world study by Yan et al. (40% ORR in prior ICI-exposed patients) (16), demonstrating that RC48 plus PD-1 blockade can overcome prior ICI resistance. This mechanism is likely driven by RC48’s bystander killing effect, which enables its cytotoxic payload to diffuse across cell membranes and exert antitumor activity on adjacent HER2-negative tumor cells. This process may further induce immunogenic cell death (ICD), thereby converting a “cold” tumor microenvironment into an immunologically “hot” one (10, 11). ICIs have been shown to enhance the antitumor efficacy of RC48, with the combination group demonstrating superior ORR (41.7% vs. 9.5%, P = 0.011) and median OS (13.2 vs. 7.1 months, P = 0.04) compared with RC48 monotherapy (16). The synergistic effect between ADCs and ICIs may amplify the immune-mediated antitumor response, which could further contribute to the favorable outcomes observed in HER2-low patients.

While the primary purpose of SRS in this case was to achieve local control of the brain metastasis, the subsequent achievement of a sustained systemic CR off therapy raises the intriguing possibility that the radiotherapy-induced tumor antigen release may have synergized with the ADC plus ICI regimen. This clinical observation is supported by a growing body of literature exploring the abscopal effect, wherein local radiation primes systemic antitumor immunity, especially when combined with ICIs (28–30). Although causality cannot be established in a single case, the temporal sequence suggests that SRS may have contributed to the favorable systemic outcome. The patient’s sustained CR off all anti-tumor therapy, after only eight months of RC48 plus camrelizumab, provides compelling evidence for the establishment of long-lasting immunological memory. This phenomenon of TFR has been well described in melanoma and lung cancer but rarely documented in gastric cancer (22, 23). The finite eight-month treatment course may have avoided chronic antigen stimulation that could lead to T-cell exhaustion, potentially selecting for a more durable memory response.

The development of grade 2 pneumonitis that resolved without corticosteroids may itself be a positive prognostic indicator, reflecting robust systemic immune activation (25). Withholding corticosteroids likely preserved ongoing antitumor immunity, as steroids can suppress T-cell function (27). Our experience suggests that close observation without steroids is a viable strategy for select patients with mild, self-limited grade 2 pneumonitis.

Based on this case and the literature review, we propose the following practical considerations: First, salvage therapy after ICI resistance: for HER2-low, MSS gastric cancer patients who progress on first-line chemo-immunotherapy, RC48 plus a PD-1 inhibitor represents a rational third-line option, with reported ORRs of 41-50% and documented ability to overcome prior ICI resistance. Second, brain metastasis management: brain metastases from gastric cancer should not be a contraindication to aggressive systemic therapy. SRS can control intracranial disease and may prime systemic antitumor immunity; timely sequencing of ADC plus ICI after SRS could achieve durable CR. Third, management of low-grade pneumonitis: for grade 2 pneumonitis without significant hypoxemia, conservative observation without corticosteroids may be considered to preserve antitumor immunity, with close monitoring for deterioration. Lastly, treatment discontinuation: patients who achieve a confirmed CR with ADC plus ICI may be candidates for treatment discontinuation with close surveillance.

Honestly, several limitations merit acknowledgment. First, as a single case, causality cannot be established, and our findings are hypothesis-generating. Second, the relative contribution of SRS versus RC48 plus camrelizumab to the CR remains unknown. Third, we lack dynamic biomarker data (e.g., serial ctDNA, T-cell receptor sequencing) that could provide mechanistic insights. Fourth, although routine imaging was performed throughout treatment, no follow-up gastroscopy was conducted to assess the primary lesion, as the patient remained free of gastrointestinal symptoms. Finally, publication bias may inflate reported efficacy estimates for ADC plus ICI combinations.

## Conclusion

In conclusion, this case demonstrates that durable, treatment-free complete remission exceeding three years is achievable, even in a patient with chemo-immunotherapy refractory gastric cancer and brain metastasis, following treatment with SRS and RC48 plus camrelizumab. The case provides clinical evidence for the ability of ADC plus ICI combinations to reverse PD-1 resistance, the potential of SRS to prime systemic anti-tumor immunity, and the feasibility of durable remission after a finite treatment course, likely mediated by immunological memory. Prospective studies incorporating dynamic biomarker analyses are urgently needed to validate these observations and identify patients most likely to benefit from this promising approach.

## Data Availability

The original contributions presented in the study are included in the article/supplementary material. Further inquiries can be directed to the corresponding author.
